# Comparative evaluation of antimicrobial properties and surface roughness of four maxillofacial prosthetic materials

**DOI:** 10.1016/j.jobcr.2025.09.019

**Published:** 2025-09-24

**Authors:** Zaihan Ariffin, Yanti Johari, Farhana Rahman, Suharni Mohamad, Nafij Bin Jamayet, James Dudley

**Affiliations:** a School of Dental Sciences, Universiti Sains Malaysia, Health Campus, Kubang Kerian, Kota Bharu, Kelantan, 16150, Malaysia; b School of Dentistry, Management and Science University, Persiaran Olahraga, Section 13, Shah Alam, Selangor, 40100, Malaysia; c Division of Restorative Dentistry, School of Dentistry, International Medical University, Jalan Jalil Perkesa-19, Bukit Jalil, Kuala Lumpur, 5700, Malaysia; d Adelaide Dental School, The University of Adelaide, South Australia, Australia

**Keywords:** Antimicrobial effect, Surface roughness, Adhesion, Silicone elastomers

## Abstract

**Objectives:**

The adherence of microorganisms to the surfaces of maxillofacial prosthetic materials can cause surrounding tissue infections leading to discomfort, irritation and infection. It is therefore beneficial if maxillofacial prosthetic materials possess antimicrobial effects or resist microbial adherence. The purpose of this study was to compare the antimicrobial effect, surface roughness and microbial adherence of a locally produced modified polymethyl methacrylate maxillofacial prosthetic material with a commercially produced polymethyl methacrylate and two silicone elastomers against three microorganisms.

**Methods:**

Four study groups were formed, each with 10 samples (n = 10): modified polymethyl methacrylate (m-PMMA), commercially produced polymethyl methacrylate (c-PMMA), silicone A-2000 (A-2000), and silicone A-2186 (A-2186). The microorganisms tested against the four prosthetic materials were *Staphylococcus aureus (S. aureus), Streptococcus mutans (S. mutans), and Candida albicans (C. albicans)*. The antimicrobial effect, microbial adherence and surface roughness were assessed and scanning electron microscopy images examined surface roughness and microbial adherence.

**Results:**

None of the tested materials showed antimicrobial activity against the evaluated microbial strains. Microbial adherence was significantly higher on silicone elastomers, with greater colony-forming units of both *S. aureus* and *S. mutans* compared to PMMA (p < 0.017). No significant difference was observed in *C. albicans* adherence between the silicone elastomers and PMMA. Surface roughness analysis revealed a statistically significant difference between PMMA and silicone elastomers (p < 0.05), with the silicones exhibiting greater roughness.

**Conclusions:**

The fillers in m-PMMA may inhibit the release of antimicrobial agents. The locally produced m-PMMA demonstrated less microbial adherence in comparison to other tested materials.

**Clinical implications:**

The locally produced m-PMMA was associated with less microbial adherence in comparison to other tested materials and has potential to reduce the risk of infection.

## 1 Introduction

Maxillofacial prostheses restore facial tissues lost due to trauma, accidents or lesions linked to precancerous or cancerous conditions in the head and neck area. These prostheses strive to achieve acceptable aesthetic outcomes and enhance the functional performance of facial muscles.^1^ The materials used to create maxillofacial prostheses must have specific properties such as durability, flexibility, high tear strength, hardness similar to natural facial tissue, colour stability, low water absorption and biocompatibility.^2^ Another important consideration in choosing materials is minimizing bacterial adhesion, as microbial colonization can lead to infections, tissue irritation, and discomfort for prosthesis users. Therefore, materials with natural antibacterial properties and limited microbial adhesion are very desirable.^2^, ^3^, ^4^ Surface topography and chemical composition greatly affect microbial adherence, with surface imperfections encouraging the growth and persistence of microbial colonies.^5^

A study conducted in dental laboratories found that 67 % of prosthetic items transferred from dental clinics were contaminated with bacteria of varying opportunistic pathogenicity.^6^ This contamination often includes blood, saliva, and oral tissues, poses a risk of transmitting infectious diseases to both dental and laboratory staff. Standard cleaning protocols for prostheses usually involve removing biofilm through manual brushing or rinsing with water and neutral soap, along with using cleansing tablets,^7^, ^8^, ^9^, ^10^ 0.5–1 % sodium hypochlorite^3^ and 2–4 % chlorhexidine. However, frequent exposure to these disinfectants can lead to negative effects.^11^ Repeated use of these cleaning agents can change the physical characteristics of silicone-based prostheses, such as increased surface roughness and decreasing hardness.^11^ The oral and facial environments provide favourable conditions for microbial colonization. When microorganisms attach to prosthetic surfaces, they produce extracellular polysaccharides that facilitate biofilm formation.^12^ Therefore, disinfectants should be chosen based on their antimicrobial efficacy, biocompatibility with surrounding tissues and minimal impact on the structural integrity of prosthetic materials.

The present study involved modifying polymethyl methacrylate (PMMA) by adding 0.5 % benzoyl peroxide (BPO), 2 % hydroxyapatite (HA) and 2 % polylactic acid (PLA) filler particles to improve its antibacterial properties against *Staphylococcus aureus (S. aureus), Streptococcus mutans (S. mutans), and Candida albicans (C. albicans).* PMMA and silicone elastomers were selected for evaluation as they are commonly used in maxillofacial prosthetics. *S. aureus* is a facultative anaerobic, gram-positive coccus found in the nasal cavity of approximately 30 % of the population, making it a significant infection risk. *S. mutans* is also a gram-positive facultative anaerobe that is common in the oral cavity and contributes to dental caries. *C. albicans* is an opportunistic fungal pathogen often linked to denture stomatitis and is frequently found on the surfaces of denture bases and silicone elastomers in denture wearers.^13^

The aim of this study was to compare the antimicrobial effect, surface roughness and microbial adherence of a locally produced modified polymethyl methacrylate maxillofacial prosthetic material with a commercially produced polymethyl methacrylate and two silicone elastomers against three microorganisms.

## 2 Materials and methods

### 2.1 Sample characteristics

The sample size was determined using PS Power and Sample Size Calculations software, where *n* represents the sample size, *δ* is the mean deviation, and *σ* represents the standard deviation. Based on estimates of σ = 0.80 and δ = 3.49, a total of 40 samples were calculated, with 10 samples allocated to each study group. The groups were Group I: Modified PMMA (m-PMMA) (n = 10); Group II: Conventional PMMA (c-PMMA) (n = 10); Group III: Silicone A-2000 (n = 10); Group IV: Silicone A-2186 (n = 10). The samples were used to evaluate antimicrobial activity, microbial adhesion, and surface roughness.

### 2.2 Sample fabrication

Following the manufacturer's instructions (Vertex, Soesterberg, The Netherlands) disc- and square-shaped samples were fabricated for each group. The materials were embedded in Type IV die stone. After the gypsum hardened, flasks containing disc-shaped samples were placed in boiling water for 10 min to remove wax. Heat-cured denture base materials for Groups I and II were mixed and packed into molds, then polymerized under 100 Hg bar pressure in a water bath at 99 °C for 2 h. Samples were retrieved using a handheld micromotor and acrylic bur, then polished with 280-grit sandpaper to a final size of 1.1 cm diameter and 0.6 mm thickness.

Silicone elastomers (Groups III and IV) were mixed by hand until they were uniform and fee of bubbles, then poured into gypsum molds. Once cured to a tack-free state, samples were trimmed to 1.1 cm diameter and 0.6 mm thickness with scissors. Square-shaped samples (10 mm × 10 mm × 2 mm) were made using the same procedures. All samples were washed, soaked in 70 % ethanol for 5 min, rinsed 3 times with sterile distilled water, and autoclaved at 121 °C for 2 h. Samples were then air-dried at room temperature for 1 week before testing.^7^^,^^14^

### 2.3 Microbial culture preparation

*S. aureus* and *S. mutans* were grown on Mueller-Hinton Blood Agar (MHBA, Oxoid, UK) at pH 7.4 ± 0.1. They were incubated aerobically at 37 °C for 24 h. *C. albicans* was grown on Sabouraud Dextrose Agar (SDA, Oxoid, UK) under the same conditions at 30 °C. After 24 h, *S. aureus* and *S. mutans* were transferred to Brain Heart Infusion (BHI) broth, while *C. albicans* was moved to Sabouraud Dextrose Broth and incubated at 30 °C for 24 h.^15^

### 2.4 Sample testing

A standardized inoculum (0.5 McFarland standard, 1.5 × 10^8^ CFU/mL) was prepared by suspending colonies in 3 mL BHI broth and vortexing. A sterile cotton swab was used to spread 100 μL of the suspension onto Mueller Hinton Blood Agar plates. Test materials were placed on the inoculated plates and incubated at 37 °C for 24 h. Distilled water was used as the negative control, and a cefuroxime disc as the positive control. Each test was performed in triplicate.^15^

Antifungal activity against *C. albicans* was measured using agar disc diffusion according to CLSI guidelines (2019). A 0.5 McFarland standard suspension was prepared in Sabouraud Dextrose broth and spread evenly on Sabouraud Dextrose Agar plates. Test materials were placed on the agar surface and incubated at 30 °C for 24 h. Nystatin and distilled water served as positive and negative controls, respectively. Each test was repeated three times.

Surface roughness was measured using a stylus profilometer (Surfcom Flex, Seimitsu Co., Ltd., Tokyo, Japan). Measurements were taken at three locations (center and edges), and mean Ra values (μm) were calculated. The profilometer settings included an evaluation length of 0.80 mm/s, cut-off value of 0.60 mm/s, and measurement speed of 0.30 mm/s. Calibration was performed before each test using a standard roughness block provided by the manufacturer.

Microbial adhesion was assessed using the method of Samaranayake and MacFarlane (1980). Stock cultures of *S. aureus*, *S. mutans*, and *C. albicans* were streaked onto Mueller Hinton Blood Agar and Sabouraud Dextrose Agar, respectively, and incubated for 24 h at 37 °C and 30 °C. Four loops of each microorganism were transferred into 100 mL of sucrose-supplemented BHI or Sabouraud Dextrose broth in centrifuge tubes. Samples were centrifuged at 2500×*g* for 10 min, and the supernatant was discarded. The sediment was resuspended in 2 mL phosphate-buffered saline (PBS) and vortexed for 10 min to reduce aggregation.^16^ Serial dilutions were prepared, and 100 μL of each dilution was plated on Mueller Hinton Blood Agar (for *S. aureus* and *S. mutans*) and Sabouraud Dextrose Agar (for *C. albicans*), then incubated for 24 h. Colony-forming units (CFU/mL) were calculated using the formula: (Number of colonies) × 10 × (reciprocal of dilution factor).^15^^,^^17^

### 2.5 Scanning electron microscopy (SEM) examination

Samples were washed 3 times with PBS and fixed in 8 % formaldehyde at 4 °C for 48 h. They were then dehydrated in graded ethanol (30 %–100 %) for 10 min at each step, followed by immersion in hexamethyldisilazane (HMDS) for 10 min and air-dryed in a desiccator. A 31.2 nm gold coating was applied using a sputter coater to minimize charging. Samples were mounted on aluminum stubs with carbon tape and examined under SEM at 10,000 × and 20,000 × magnifications.

### 2.6 Statistical analysis

All statistical analyses were performed using SPSS version 22 (IBM, USA). The Shapiro-Wilk test assessed data normality. One-way ANOVA was used to compare surface roughness among groups, with a significance threshold of *p* < 0.05. Microbial adhesion data were analyzed using multivariate analysis of variance (MANOVA), with significance set at *p* < 0.017.

## 3 Results

### 3.1 Antimicrobial activity

**Fig. 1:**
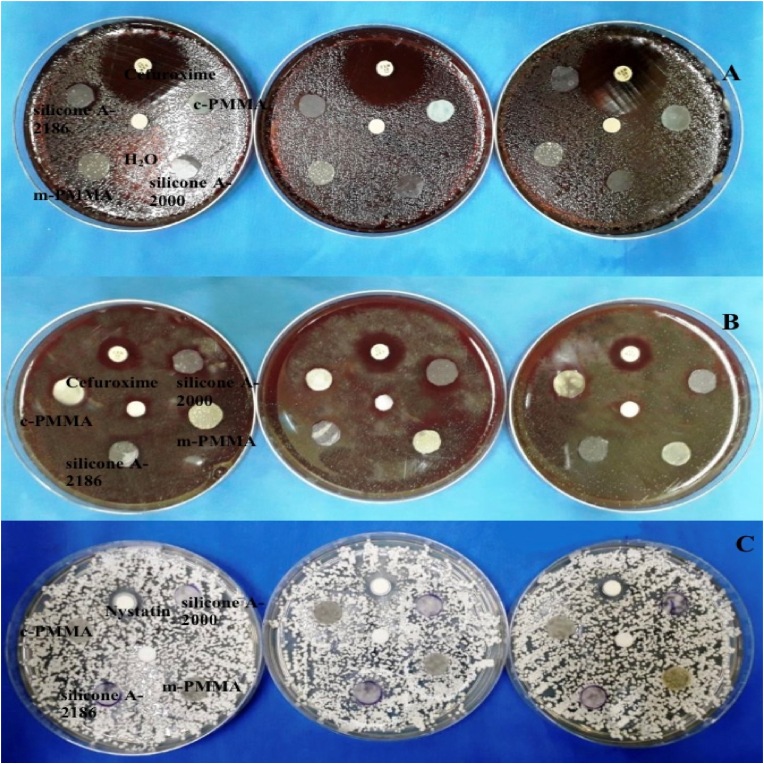
Agar diffusion tests of PMMA and silicone elastomers against (A) *S. aureus* (B) *S. mutans* and (C) *C. albicans*.

The antimicrobial activity of PMMA and silicone elastomers was evaluated based on the presence or absence of inhibition zones. After 24 h of incubation, none of the tested materials exhibited any inhibition zones against the bacterial or fungal strains (Fig. 1). In contrast, the positive controls, cefuroxime (antibacterial) and nystatin (antifungal), demonstrated clear zones of inhibition, confirming their efficacy. The negative control (distilled water) showed no inhibitory effect on any of the tested microorganisms (Table 1).

**Table 1:** Antimicrobial activity of maxillofacial prosthetic materials and control groups.

Material	Microorganisms – Mean zone of Inhibition (mm)		
	Staphylococcus aureus	Streptococcus mutans	Candida albicans
Study group I (m-PMMA)	No	No	No
Study group II (c-PMMA)	No	No	No
Study group III (silicone A-2000)	No	No	No
Study group IV (silicone A-2186)	No	No	No
Positive Control for Bacteria (Cefuroxime)	30.39	15.36	–
Negative Control for Candida (Nystatin)	–	–	17.56
Negative Control (dH_2_O)	No	No	No

m-PMMA - Modified Polymethyl Methacrylate; c-PMMA - Commercially produced Polymethyl Methacrylate; dH_2_O - distilled Water; ‘No’ indicates no inhibition zone.

### 3.2 Surface roughness

The surface roughness analysis revealed that silicone A-2000 and silicone A-2186 had significantly rougher surfaces compared to m-PMMA and c-PMMA. No statistically significant difference was observed between m-PMMA and c-PMMA (p > 0.978). However, m-PMMA showed significantly smoother surfaces compared to silicone A-2000 (p < 0.000) and silicone A-2186 (p < 0.003) (Table 2).

**Table 2:** Comparison of mean surface roughness of m-PMMA and commercially available biomaterials Mean, SD (Ra, μm).

Biomaterial (Mean ± SD) (Ra, μm)		Surface Roughness (Mean ± SD) (Ra, μm)	P Value
Study group I (m-PMMA) (1.804 ± 0.359)	Study group II (c-PMMA)	(1.718 ± 0.192)	0.978
	Study group III (Silicone A-2000)	(3.153 ± 0.635)	<0.001∗∗∗
	Study group IV (Silicone A-2186)	(2.622 ± 0.592)	0.003∗∗

m-PMMA - Modified Polymethyl Methacrylate; c-PMMA - Commercially produced Polymethyl Methacrylate; ∗∗P < 0.01; ∗∗∗P < 0.001.

Level of significance set at p < 0.05, using one-way ANOVA test.

### 3.3 Surface topography (SEM)

**Fig. 2:**
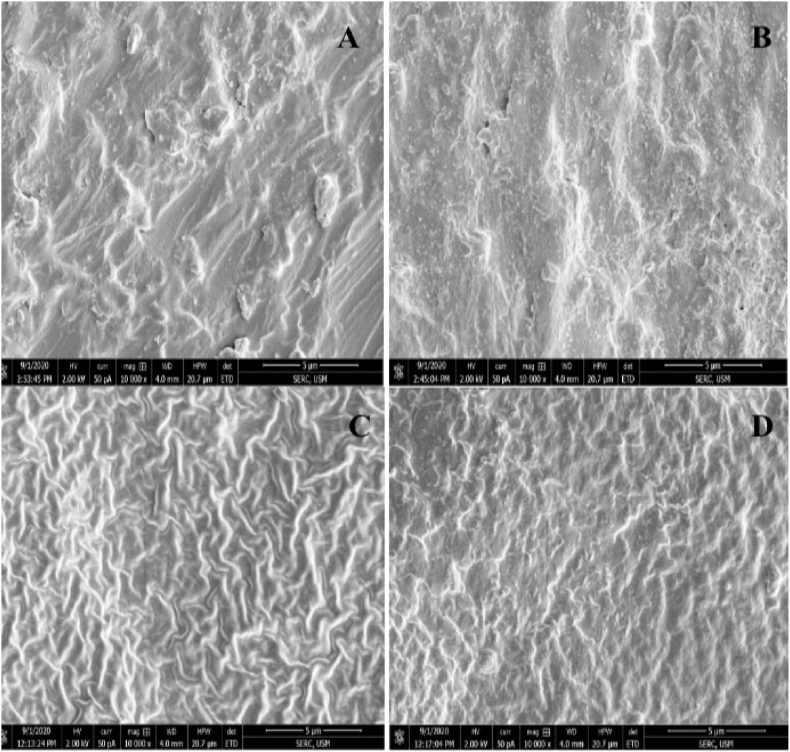
SEM images of maxillofacial prosthetic materials (10,000x magnifications) (A) m-PMMA, (B) c-PMMA, (C) silicone A-2000, (D) silicone A-2186.

SEM analysis at 10,000 × magnification showed clear differences in surface topographies among the materials. PMMA samples (both m-PMMA and c-PMMA) had fewer, irregularly distributed surface elevations. In contrast, silicone elastomers (A-2000 and A-2186) displayed more uniformly distributed surface features (Fig. 2).

### 3.4 Microbial adherence

**Fig. 3:**
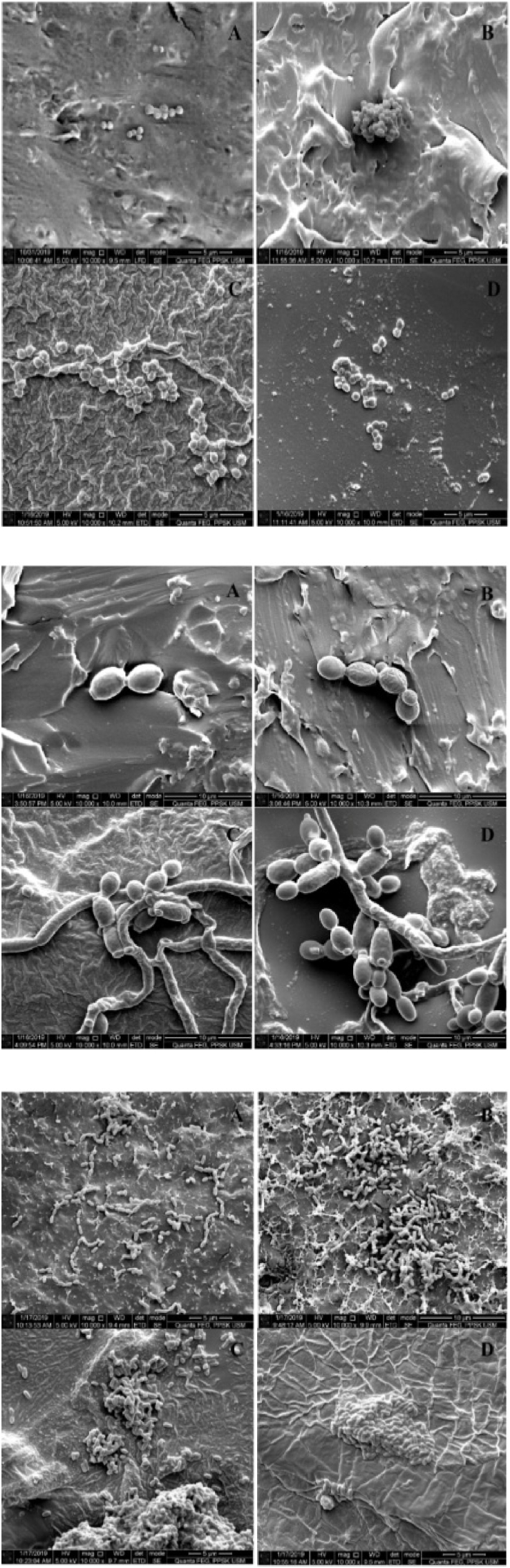
SEM images of *S. aureus*, *S. mutans and C. albicans* adherence on maxillofacial prosthetic materials after 24 h incubation (10,000x magnifications) (A) m-PMMA, (B) c-PMMA, (C) silicone A-2000, (D) silicone A-2186.

Microbial adherence of *S. aureus*, *S. mutans*, and *C. albicans* to the tested materials after 24 h is shown in Table 3 and visualized in Fig. 3. *S. aureus* adhered significantly less to m-PMMA compared with silicone A-2000 (p < 0.014) and silicone A-2186 (p < 0.002). No significant difference was observed between the two silicone elastomers. Similarly, *S. mutans* adherence was significantly reduced on m-PMMA compared with silicone A-2000 (p < 0.006) and silicone A-2186 (p < 0.004). In contrast, *C. albicans* adherence did not show any significant difference among the four tested materials.

**Table 3:** Pairwise comparison of mean differences of m-PMMA (CFU/mL) and commercially available tested biomaterials.

Microorganism (CFU/mL)	Biomaterials (Mean ± SD × 10^13^)	Biomaterials (Mean ± SD × 10^13^)	P Value
*Staphylococcus aureus*	Study group I (m-PMMA) (1.733 ± 0.115)	study group II (c-PMMA) (2 ± 0.200)	0.111
		study group III (silicone A-2000) (2.2 ± 0.200)	0.014∗
		study group IV (silicone A-2186) (2.4 ± 0.200)	0.002∗∗
*Streptococcus mutans*	Study group I (m-PMMA) (1.866 ± 0.305)	study group II (c-PMMA) (1.8 ± 0.200)	0.694
		study group III (silicone A-2000) (2.46 ± 0.115)	0.006∗∗
		study group IV (silicone A-2186) (2.533 ± 0.115)	0.004∗∗
*Candida albicans*	Study group I (m-PMMA) (2.813 ± 0.140)	study group II (c-PMMA) (2.78 ± 0.180)	0.863
		study group III (silicone A-2000) (2.76 ± 0.262)	0.731
		study group IV (silicone A-2186) (2.73 ± 0.115)	0.608

Note: m-PMMA - Modified Polymethyl Methacrylate; c-PMMA - Commercially produced Polymethyl Methacrylate; ∗P < 0.05; ∗∗P < 0.01.

Level of significance set at p < 0.05, using MANOVA test.

### 3.5 SEM analysis of microbial morphology

SEM images at 10,000 × magnification showed *S. aureus* as round, grape-like clusters, especially noticeable on silicone surfaces. *S. mutans* appeared mainly in diplococci form, with less clustering on PMMA surfaces. *C. albicans* cells were large, round structures with hyphal extensions. Hyphal formation was more noticeable on silicone elastomers (A-2000 and A-2186) than on PMMA materials (m-PMMA and c-PMMA), although these differences were not statistically significant.

## 4 Discussion

The present study provides valuable insight into the antimicrobial effect, surface roughness and microbial adherence of a locally produced modified polymethyl methacrylate maxillofacial prosthetic material compared with a commercially produced polymethyl methacrylate and two silicone elastomers against three microorganisms. The research addresses an important challenge posed by these materials. Antimicrobial activity and microbial adherence are critical factors in selecting materials for maxillofacial prostheses, as these devices are in direct contact with skin and mucosa making them vulnerable to microbial colonization and infection.^9^^,^^18^ PMMA and silicone elastomers are commonly used due to their affordability, ease of handling, and favourable mechanical properties. However, their susceptibility to microbial adhesion requires management through incorporating antimicrobial agents, mechanical cleaning, applying chemical disinfectants or modifying the material itself.^19^, ^20^, ^21^

This study used the agar-disc diffusion method which is an economical, reproducible and widely accepted technique for evaluating antimicrobial activity against both Gram-positive and Gram-negative organisms.^8^^,^^22^^,^^23^ None of the tested materials (m-PMMA, c-PMMA, silicone A-2000, and silicone A-2186) exhibited inhibition zones after 24 h, indicating a lack of inherent antimicrobial activity (Table 1). Although m-PMMA was modified with HA and BPO, both known for their potential antimicrobial effects,^24^, ^25^, ^26^ the concentrations used may have been insufficient to produce measurable inhibition. This supports earlier findings that emphasized the importance of adequate antimicrobial agent concentration for efficacy.^27^^,^^28^

Surface roughness plays a key role in microbial colonization.^5^^,^^29^, ^30^ In the present study, PMMA samples exhibited significantly smoother surfaces than silicone elastomers likely due to differences in material composition and the polishing process. SEM analysis confirmed that PMMA surfaces had fewer elevations that were unevenly distributed compared to the more uniformly textured silicone surfaces (Fig. 2). Despite the addition of fillers, m-PMMA showed no significant change in surface roughness compared to c-PMMA, suggesting that the fillers did not alter the surface profile.

Microbial adhesion varied between materials. *S. aureus* and *S. mutans* adhered significantly less to m-PMMA than to silicone elastomers (Table 3, Fig. 3) consistent with previous studies that found higher microbial retention on silicone surfaces.^15^^,^^17^^,^^31^, ^32^, ^33^, ^34^, ^35^, ^36^ This may be attributed to the smoother, polished PMMA surfaces that reduce bacterial retention. In contrast, *C. albicans* adherence did not significantly differ among the materials, suggesting that its adhesion may be influenced less by surface roughness and more by factors such as surface chemistry or receptor-mediated interactions. Microbial adhesion is governed by complex physicochemical interactions including surface roughness, hydrophobicity, surface free energy, and wettability.^5^^,^^37^^,^^38^ These properties are closely linked to the polymer's composition and can be altered by the release of components into the surrounding environment.^33^ Rougher surfaces and higher surface charges tend to promote microbial attachment, while increased wettability can reduce it. Adhesion typically occurs in two stages: initial physicochemical interaction followed by specific receptor-mediated binding.^39^ In this study, m-PMMA was modified with HA, PLA and BPO to enhance mechanical strength and potentially influence surface chemistry. HA and PLA were added to improve flexural strength and impact resistance respectively, while BPO served as a polymerization initiator.^40^ The presence of HA and BPO may have contributed to reduced bacterial adherence on m-PMMA due to its known antimicrobial properties.^41^^,^^42^ Although no inhibition zones were observed, the lower adherence of *S. aureus* and *S. mutans* to m-PMMA suggests a possible antibacterial effect. This aligns with findings that HA can reduce bacterial adhesion through surface modification,^41^ and that BPO generates reactive oxygen species that disrupt bacterial membranes.^42^

Surface energy and charge also play a role in microbial adhesion.^34^ The addition of fillers may have altered the surface energy of m-PMMA, therefore reducing bacterial affinity. In contrast, silicone A-2000 and A-2186, which likely share similar surface energies, showed higher bacterial adherence. Wettability further explains these differences. PMMA is hydrophilic with higher wettability, while silicone is hydrophobic with less wettability.^43^ The reduced adherence of *S. aureus* and *S. mutans* to m-PMMA is consistent with previous studies showing increased hydrophilicity lowers microbial attachment.^34^ *S. aureus*, a coagulase-negative staphylococcus with hydrophobic properties, adheres more readily to hydrophobic silicone surfaces.^44^, ^45^ No significant difference in bacterial adherence was observed between the two silicone types, suggesting similar surface characteristics.

Microbial adhesion is also species-specific and influenced by surface topography. SEM images revealed *S. aureus* formed grape-like clusters and *S. mutans* appeared as diplococci, both more densely packed on silicone than PMMA surfaces. *C. albicans* exhibited hyphal growth, particularly on silicone, though no significant differences in fungal adherence were found. Larger fungal cells may detach more easily from smoother PMMA surfaces during the washing phases required to prepare samples for SEM examination.^46^, ^47^, ^48^

Surface roughness was measured using a stylus profilometer which is a practical and widely used tool for material analysis.^49^ While atomic force microscopy offers higher resolution and operates in ambient environments,^50^ the stylus profilometer provided sufficient data for this study. PMMA samples showed smoother surfaces than silicone, correlating with reduced bacterial adhesion. These findings highlight the utility of stylus profilometry in detecting clinically relevant surface differences that may influence microbial interactions with prosthetic materials.

This in vitro study was limited by the fixed concentrations of HA (2 %) and BPO (0.5 %) in m-PMMA, which may have been insufficient to elicit measurable antimicrobial effects as evidenced by none of the materials exhibiting direct antimicrobial activity against *S. aureus, S. mutans* or *C. albicans*. The study was limited to three of the common microorganisms and conducted in a controlled in vitro environment. Although in vitro antimicrobial results demonstrated potential efficacy, extrapolation to clinical outcomes should be approached with caution, as further in vivo investigations and controlled clinical studies are necessary to substantiate these findings within patient contexts. Future research should explore alternative antimicrobial agents or higher filler concentrations in m-PMMA formulations. Broader microbial testing and in vivo studies are also recommended to better understand the clinical implications of material modifications. Continued investigation is critical before any definitive conclusions regarding clinical efficacy can be drawn. Further research may highlight the promising potential of m-PMMA as a modifiable biomaterial in maxillofacial prosthetic applications.

## 5 Conclusions

Within the limitations of this in vitro study, the fillers in m-PMMA may inhibit the release of antimicrobial agents. The locally produced m-PMMA demonstrated less microbial adherence in comparison to other tested materials and has potential to reduce the risk of infection. These findings highlight the influence of surface characteristics on microbial colonization and underscore the need for further material modifications to enhance antimicrobial performance in maxillofacial prostheses.

Ethics approval was not required for this study.

Patient's/Guardian's consent approval was not required for this study.

## Credit statement

Conceptualisation: ZA, YJ, FR, SM, NJ, JD.

Methodology Design: ZA, YJ, FR, SM, NJ, JD.

Data Collection: YJ, FR, SM.

Data Processing: YJ, FR, SM.

Metadata synthesis: ZA, YJ, FR, SM.

Data Interpretation: YJ, FR, SM, NJ, JD.

Manuscript writing: ZA, YJ, FR, SM, NJ, JD.

Manuscript revision: ZA, YJ, FR, SM, NJ, JD.

Final Approval for Submission: ZA, YJ, FR, SM, NJ, JD.

All authors have read and agreed to the published version of the manuscript.

## Funding

This study was supported by Universiti Sains Malaysia Research University Grant No 1001/PPSG/8012380.

## Declaration of competing interest

The authors declare that they have no known competing financial interests or personal relationships that could have appeared to influence the work reported in this paper.
